# Model Driven Development Applied to Complex Event Processing for Near Real-Time Open Data

**DOI:** 10.3390/s18124125

**Published:** 2018-11-24

**Authors:** Pedro J. Clemente, Adolfo Lozano-Tello

**Affiliations:** Quercus Software Engineering Group, Instituto de Investigación en Tecnologías Aplicadas de Extremadura (INTIA), University of Extremadura, 06071 Badajoz, Spain; alozano@unex.es

**Keywords:** open data, complex event processing, model-driven development, model to text transformation, data analysis

## Abstract

Nowadays, data are being produced like never before because the use of the Internet of Things, social networks, and communication in general are increasing exponentially. Many of these data, especially those from public administrations, are freely offered using the open data concept where data are published to improve their reutilisation and transparency. Initially, the data involved information that is not updated continuously such as budgets, tourist information, office information, pharmacy information, etc. This kind of information does not change during large periods of time, such as days, weeks or months. However, when open data are produced near to real-time such as air quality sensors or people counters, suitable methodologies and tools are lacking to identify, consume, and analyse them. This work presents a methodology to tackle the analysis of open data sources using Model-Driven Development (MDD) and Complex Event Processing (CEP), which help users to raise the abstraction level utilised to manage and analyse open data sources. That means that users can manage heterogeneous and complex technology by using domain concepts defined by a model that could be used to generate specific code. Thus, this methodology is supported by a domain-specific language (DSL) called OpenData2CEP, which includes a metamodel, a graphical concrete syntax, and a model-to-text transformation to specific platforms, such as complex event processing engines. Finally, the methodology and the DSL have been applied to two near real-time contexts: the analysis of air quality for citizens’ proposals and the analysis of earthquake data.

## 1. Introduction

Currently, many companies and public administrations are adopting the open data paradigm in order to offer transparent information such as contracts, budgets, resources, and so on [1]. Specifically, open-data publishing is an emerging trend for public administrations [2] and smart cities [3,4]. For them, data reutilisation and transparency are key elements around which a new economy based on the value of data could be defined [5,6]. The main benefits of open data [1] include (i) political and social benefits, such as more transparency, democratic accountability, more participation and self-empowerment of citizens, public engagement or scrutinisation of data; (ii) economic benefits, such as economic growth and stimulation of competitiveness, stimulation of innovation, development of new products and services, creation of a new sector adding value to the economy; and (iii) operational and technical benefits, such as the ability to reuse data, optimisation of administrative processes, fair decision-making by enabling compassion, the creation of new data based on combining data, external quality checks on data (validation), and the ability to merge, integrate and mesh public and private data. In general, the main consequences for citizens are that using public data could help them to understand and analyse what is happening around them in real-time.

Complex Event Processing (CEP) [7,8] is an approach that is used to carry out real-time complex analyses based on event pattern identification. Thus, raw data from the external world are notified as *events* which can be filtered, combined, or correlated in order to identify *event patterns* that are given to the interested parties [9]. CEPs have been used successfully in several areas, such as healthcare, home automation, operational intelligence in business and transportation, and traffic management. Usually, in order to carry out this kind of analysis, CEP engines offer a stream-oriented language which extends SQL [8], for instance, StreamSQL in StreamBase [10] or EPL in Esper [11] include filtering, combining, or temporal window primitives [12,13].

Nowadays, many of the open data producers offer data in real-time [4], so a real-time analysis of open data could improve their use by third parties (citizens, external companies, other administrations, etc.). For instance, a data user can analyse the *pH* value of the water and identify unusual data values in real-time. These data could be used as inputs to CEP engines. On the other hand, open data producers offer data using well-known file formats such as CSV, Excel, PDF, XML (https://www.w3.org/XML/), JSON (http://json.org) or RDF (https://www.w3.org/RDF/). Thus, users can query the data catalogues in open-data websites or download the data in common file formats such as CSV, Excel, or PDF. Usually, data formats such as XML, JSON or RDF are not downloaded directly, but also, they are consumed by using specific software tools or mobile apps. Professional users can design complex queries using SparQL [14] a query language upon RDF format information. However, defining and analysing open data is highly complex process and, as a consequence, open data sources could be difficult for final users to analyse.

On the other hand, many data catalogues can be continuously updated (real-time publishing), for instance, the pollution data gathered from pollution sensors distributed throughout a city. In order to manage continuous queries where the data is published using RDF, the W3C RDF Stream Processing (RSP) Community Group (https://www.w3.org/community/rsp/) aims to define a common model for producing, transmitting, and continuously querying RDF streams, including extensions to both RDF and SPARQL to represent streaming data as well as their semantics. Thus, several proposals could be used to carry out continuous queries on RDF sources such as C-SPARQL [15] or TripleWave [16]. However, on the one hand, data endpoints based on SparQL are uncommon nowadays because open data information is usually offered using more common data formats, such as CSV, JSON and specific APIs. On the other hand, general users and unprofessional users (for instance, citizens), in particular, require methodologies and tools that improve the access and analysis of continuous data catalogues (data streams) available in open data sources not only in RDF but also in JSON or CSV.

In order to tackle this heterogeneous technology (complex event processing engines, open data tools, notification technology, etc.) we used model-driven development [17,18,19,20] increases the abstraction level where the software is developed, focusing on the domain concepts and their relationships. These concepts and relationships are defined by a model which, for instance, can be analysed and validated before automatically generating code. Besides, text code can be generated from a model using model-to-text transformation, decreasing the incidence of user errors while increasing the user productivity. Thus, in the context of model-driven development, software development is guided through Models (M1) which conform to a MetaModel (M2). Further, a Metamodel conforms to a MetaMetaModel (M3) which is reflexive [21]. The MetaMetaModel level is represented by well-known standards and specifications, such as Meta-Object Facilities (MOF) [22] and ECore in EMF [23]. A MetaModel defines the concepts and relationships in a specific domain in order to model partial reality. Then, these models can be used to totally or partially generate the application code by model transformations [24]. Thus, the software code can be generated for a specific technological platform, improving the technological independence and decreasing the error proneness.

This study proposes a methodology to (a) identify data catalogues in open data sources; (b) define complex patterns to be analysed in the data catalogues; and finally (c) define how results should be notified using several kinds of formats, such as email, Twitter, JMS, REST API, etc. Additionally, a Domain-Specific Language is developed using model-driven development techniques, supporting the aforementioned methodology that allows users to define and to carry out complex and continuous analyses on real-time open data. Besides, in order to integrate heterogeneous technology, an Enterprise Service Bus (ESB) [25] is used to deploy the system generated.

The main contributions of this study are as follows:

- This paper shows that model-driven development is a suitable approach to the development of tools to tackle the complexity of heterogeneous technology as occurs in the context of open data sources and complex event processing.
- A methodology named OpenData2CEP is defined to describe each step needed to carry out the analysis of complex event patterns on open data sources.
- A model-driven approach is developed to support the methodology proposed. It facilitates the development of each methodological phase by defining an *OpenData2CEP* metamodel and a model-to-text transformation towards code generation for a specific Enterprise Service Bus (ESB) and CEP engine which facilitate the deployment of the analysis defined.
- Two case studies are developed following the methodology and tools presented which include different event pattern analyses.

The rest of the paper is structured as follows. In Section 2, we give an overview of existing open data tools, stream processing engines and languages, and model-driven approaches that are applied to open-data and complex event processing. In Section 3, we present the *OpenData2CEP* methodology. Section 4 describes the *OpenData2CEP* design and implementation phases including the *OpenData2CEP Metamodel*. In Section 5, two case studies are presented. Finally, Section 6 elaborates on the limitations of the present approach before Section 7 concludes the paper.

## 2. Related Works

The present study unites two well-known research areas: open data and stream processing. They are tackled from the model-driven point of view; as a consequence, this section reviews general open data analysis tools, stream processing approaches, and model-driven studies related to open data and stream processing.

### 2.1. Open Data Tools

Currently, several tools can be found that define, publish, and visualise open-data. Common tools with the aim of defining open data catalogues include Open Refine [26] (formerly Google Refine), Socrata [27], CKAN [28], Protégé or Google Fusion Tables [29]. Open Refine [26] makes it possible to work with messy data by cleaning them, transforming them from one format into another, aligning data with linked-data databases, etc. Regarding the inclusion of semantic structure in open data using RDF format, the most used tools is Protégé [30]. CKAN [28] is a data management system that is focused on publishing, sharing, finding, and using data. Currently, it is used by data publishers, such as national and regional governments, companies and organisations, and can offer open data catalogues with several file formats, such as PDF, CSV, XML, RDF, etc. Google Fusion Tables [29] focus on how to manage and visualise data catalogues, making it possible create charts, maps, network graphs from data catalogues with hundreds of thousands of rows. Finally, Socrata [27] is one of the most important platforms for publishing and managing open data, and is especially used by USA governments including the federal government, state governments, county governments, or city governments.

Regarding the use of ontology-based software tools for open data in the Internet of Things (IoT) domain, there are several projects such as *SSN Ontology Validation Service* [31], which was designed in the context of the CityPulse FP7 EU project to validate the RDF dataset designed according to the SSN V1 ontology; *Read4SmartCities* [32], a project providing a catalogue of ontologies relevant for building smart cities focused on different domains (energy, climate, weather, environment, building, occupancy, user behaviour and characteristics); *OpenSensingCity* [33], which provides a web portal that collects information about the smart cities and provides web applications to visualise the list of existing projects, ontologies, and datasets; or *Linked Open Vocabularies for the Internet of Things (LOV4IoT)* [34], which references 391 ontologies related to an IoT applicative domain exploiting sensor and/or semantic web technology. There are collections and analyses of more open data and IoT projects and software tools in [35,36,37].

These tools make it possible to define, publish, visualise, query, and filter open data catalogues; however, they are not designed to analyse stream data.

### 2.2. Stream Processing Languages and Engines

Nowadays, event stream processing is an emergent area that has the goal of managing the intensive data produced from smart cities, smart agro, system interactions, etc., in order to analyse and correlate them with the aim of identifying important and critical situations. In this context, stream processing engines that manage huge volumes of data over short periods of time require efficient mechanisms to analyse, query, and correlate data.

Event processing can be addressed by Event Stream Processing (ESP) and Complex Event Processing (CEP). On the one hand, events can be processed in order by stream processing which takes into account the timestamp when the events are produced. So, this kind of data can be managed using Event Stream Processing (Apache Storm [38], Apache Samza [39] or S4 [40]), which can process event streams using very little memory because they do not have to remember many events. However, if we want to carry out a complex analysis based on identifying event patterns, a CEP (Complex Event Processing) engine should be used. In this sense, CEP is a superset of ESP [13,41].

Several Complex Event Processing engines have appeared with different approaches to analyse complex event patterns in stream data. Some of them define a language similar to SQL, known as an Event Processing Language (EPL), which includes temporal relations and data windows, for example Esper EPL [11], Oracle EPL [42], Stream SQL [10] or CCL [43]. Other approaches, such as Apache Kafka [44], Apache Flink [45] and FiwareCEP [46] (which is part of a wide European project named FIWARE [47]), make it possible to analyse streamed data, although they do not provide a query language like EPL, but rather, provide a wide API to carry out the Complex Event Processing.

The main characteristics of an EPL language are that it facilitates the definition of Complex Event Processing, including filters, aggregations, groups, event correlations, temporal relations, data windows, etc. In addition, the fact that the query language is similar to SQL helps users to learn and use it. In  [48] the main characteristics of popular Event Processing Languages were analysed by designing an abstract metamodel representing an EPL language. This means that Esper EPL [11], Oracle EPL [42], Stream SQL [10] or CCL  [43] query languages have core concepts that define how to analyse complex event patterns.

EPL languages include defining event patterns, which are patterns that combine other events. These event patterns were classified in [49] as including the following types: (i) *selection patterns*, which make it possible to create complex events every time a given simple event is detected; (ii) *windows*, which make it possible to assign *windows* to patterns, restricting their scope; (iii) *temporal sequencing of events* based on the operator *followedBy* (‘->’) which defines a temporal ordering between events; (iv) *pattern combinations*, where event patterns can be combined using logical operators (OR, AND, etc.) and temporal connectors such as (until or while); and (v) *high-order complex events*, which are defined as a pattern that specifies the event makes use of other complex events previously defined, for instance, using operators such as *every* or *every-distinct*.

Otherwise, Apache Kafka [44], Apache Flick [45] and FiwareCEP [46] make it possible to manage stream data that can be efficiently analysed, including filters, aggregations, joins, window data and so on. However, they do not provide a query language like EPL, but rather, they provide a wide API to carry out the Complex Event Processing. These wide API allow users to develop code in order to connect several kinds of data sources or data streams, which can be consumed using several kinds of formats (binary, XML, JSON, CSV, etc.). FiwareCEP [46] merits special attention because it offers a native mechanism to process streams coded in JSON.

Finally, in the context of linked data, that is, semantic data sources which are usually defined through RDF [50], there is a consolidated query language named SparQL [14]. However, it has an important limitation because queries are executed once and at a specific moment, and as a consequence, it does not allow processing of stream data. An extension of SparQL, named C-SparQL [15] improves the stream reasoning for linked data. It includes clauses to manage temporal relations and data windows, as do EPLs.

All of these proposals could be employed by professional users to define and execute Complex Event Processing. However, on the one hand, a deep knowledge about specific code language and specific API is needed and, on the other hand, they do not offer simple mechanisms for inexpert users to explore and improvetheir use.

### 2.3. Model-Driven Approach Applied to Open-Data and Complex Event Processing

Model-Driven technologies have been applied as a transversal resource in order to manage data and their transformation from a high abstraction level. This means that data is brought into the model-driven technological space including model, text-to-model transformations (t2m), model-to-model transformations (m2m) and model-to-text transformations (m2t). The following allow the management and analysis of data from several points of view, from event definitions to code generation, which can be executed under a specific framework.

Medit4CEP [51] uses model-driven techniques to facilitate the definition of complex event patterns which could be analysed using a CEP. It implements a graphical concrete syntax for Eclipse IDE to define complex event patterns. For this, Medit4CEP generates a second specific graphical tool for Eclipse from the data event specification which can also be defined in the first Eclipse graphical editor. Then, users can model complex event patterns using concepts similar to the ones defined in the EPL language [48]. Finally, a model-to-text transformation to the Mule ESB project is generated to manage the kind of events defined on a Complex Event Engine. However, this approach does not manage open data sources, but rather, the data is defined ad hoc through connections with previous data sources.

On the other hand, Modisco [52] is a generic and extensible framework for model-driven reverse engineering. It allows users to obtain models from text artefacts such as XML, SQL, COBOL, etc.). Specific discoverers/injectors can be implemented to extend the original Modisco approach in order to cover additional text formats. Modisco has been used successfully to obtain Schemas in JSON Data  [53] and to obtain conceptual models from legacy web applications [54]. Modisco’s flexibility could be applied to produce models from data stored in text documents.

Segura et al. [55] proposed an adaptation of the Data as a Service (DaaS) paradigm to develop open data applications. For this, model-driven engineering is used to manage the heterogeneous sources and then to publish the data using a REST API infrastructure. Specifically, multi-level modelling for the description of domains is improved based on generic meta-models and a library of injectors is offered to bring data on demand from heterogeneous sources into the MDE technical space. Finally, a REST-infrastructure is generated to access the back-end data. However, although this process makes it possible to model data, the authors did not offer a mechanism to analyse the data published in real-time.

Our approach focuses on specific concepts that can be managed from models that, to our knowledge, has not been used previously, that is, model-driven techniques are applied to the management of open data sources are then analysed by Complex Event Processing.

## 3. OpenData2CEP Methodology

**Figure 1:**
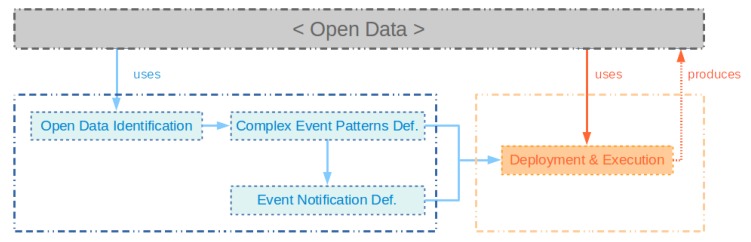
OpenData2CEP methodology overview.

In this section, we describe a methodology named *OpenData2CEP* which is able to connect open data sources to a complex event process engine in order to carry out complex analyses. The methodology includes the following phases: (a) open data source identification; (b) definition of complex event patterns using the data published from the open data catalogue; (c) definition of the notification system where an event pattern will be identified; and (d) execution of the continuous queries on the open data source. In Figure 1, the main steps, inputs, and outputs of our methodology are defined. Each one is described below.

### 3.1. Open Data Catalogue Source Identification

The identification of data sources is a process that each user should carry out by hand as each potential user will have their own interests, so they should search where their target data are published. In order to achieve this goal, users can search, for instance, on national open data sources, such as http://datos.gob.es, http://www.data.gouv.fr, http://www.dati.gov.it, http://www.data.overheid.nl, https://www.data.gov, https://usopendata.org or http://data.gov.uk, among others. As an example, much of this information can be produced in real-time, such as data gathered by pollution sensors located on city streets. For us, these frequently updated near real-time data are the most interesting data catalogues.

The data catalogue formats available are related to the concept of five stars of data reutilisation  [56]. In this sense, open data sites with five stars, that is those with the highest data reutilisation, define linked-data based on RDF, making it possible to carry out complex queries using SparQL [14]. On the other hand, open data sites identified as having four stars publish their information using RDF, and finally, common open data sites with three stars publish data by using non-proprietary data forms, such as XML, CSV, or JSON formats. Usually, this information is well organized and an endpoint (for instance, an URL) allows it to be downloaded. In any case, users should identify the URL where the data can be queried or downloaded.

As an example, the Gijón (Spain) open data website https://transparencia.gijon.es produces, as well as other information, an open data catalogue about air quality. These data are obtained from a set of sensors throughout the city and are updated every 60 min. As a consequence, for example, a user can analyse the evolution of the air quality in their home zone. In this case, the open data source, identified in this work as *AirDataG*, is published in several formats, such as TEXT, CSV, and XML formats. In addition, a PDF file describes each field in the open data source. For instance, an air quality record includes the following fields: *station, title, latitude, longitude, solar date, SO*_2_ *(sulfur dioxide), NO (nitric oxide), CO (carbon monoxide), PM10 (particulate matter 10 or PM*_10_*), O*_3_ *(ozone), dd, vv, TMP, HR, PRB, RS, LL, BEN, TOL, MXIL, PM25 (particulate matter 2.5 or PM*_2.5_*)*. Usually, this information can be obtained from the CSV header row or the data catalogue description.

### 3.2. Defining Complex Event Patterns Using the Data Published from the Open Data Catalogue

As mentioned, users of a specific data source should be able to define thecomplex event patterns which will be used to analyse the data source. For instance, in a pollution environment context, a complex event pattern could analyse several values in order to identify patterns which define risky situations. So, the complex event patterns should be defined ad-hoc by the user. Usually, a complex event pattern in the context of CEP is defined by using an Event Processing Language (EPL), which is a declarative language that deals with high frequency time-based event data.

An EPL allows developers to define complex sentences by identifying statistics and window time clauses over a dataset. For instance, the regional government has defined several limits and target values in order to maintain a suitable air quality. For instance, ozone ($O_{3}$) should have a value under 240 µg/m${}^{3}$ and SO${}_{2}$ (sulphur dioxide) should have a value under 350 µg/m${}^{3}$. Other air measures, such as $PM_{2.5}$ and $PM_{10}$, should have values based on daily and annual averages. The former, $PM_{2.5}$, should have an annual average value of less than or equal to 10 µg/m${}^{3}$ and a daily average of less than or equal to 25 µg/m${}^{3}$. The latter, $PM_{10}$, should have an annual average value of less than or equal to 20 µg/m${}^{3}$ and a daily average of less than or equal to 50 µg/m${}^{3}$. So, a flexible language should be used to define these kinds of analyses.

An EPL allows developers to define complex event patterns in order to identify statistics, time correlations, and data window clauses over a dataset. For instance, in a simple EPL query (similar to SQL), we can issue the *AirDataG* events which present risks for people:

$$\mathrm{select}*\mathrm{from}\;\mathrm{AirDataG}\;\mathrm{where}\;O_{3}>240\;\mathrm{and}\;SO_{2}>350.$$

In the previous EPL sentence, each *AirDataG* row analysed is an event. So, an event can be defined from a set of data which represents a concept. Usually, each event has several attributes that can be analysed. Thus, the SQL concepts of correlation through joins, filtering, and aggregation through grouping can be effectively leveraged. For example, in Algorithm 1, from the data event identified, a data correlation can be found where *four* events of *AirDataG* have values of O3 with a positive tendency and near to the limit established; as a consequence, an alert can be issued. In Algorithm 1, the complex event pattern is named *Air03Tendency*.

**Algorithm 1:** Example of Event Processing Language (EPL) expression to identify $O_{3}$ tendency values.
 1
**create schema** AirDataG (Station Float,Title String,Latitude Float,Longitude Float,SolarDate
 2
String, SO2 Float,NO Float,CO Float,PM10 Float,O3 Float,dd Float,vv Float,TMP Float,HR Float,
 3
PRB Float,RS Float,LL Float,BEN Float,TOL Float,MXIL Float,PM25 Float);
 4
 
 5
@Name("Air03Tencency")
 6
**select** a4.*
 7
  **from pattern** [(**every** (a1 = AirDataG(a1.O3 > 200)
 8
    -> a2 = AirDataG(a2.O3 > a1.O3)
 9
    -> a3 = AirDataG(a3.O3> a2.O3)
10
    -> a4 = AirDataG((a4.O3 > a3.O3)
11
  )))]
12
;



Therefore, EPL is an expressive language that is used to define complex queries on continuous event streams. Users should manage the common issues when dealing with the definition of complex event patterns including: data source selection, parameter selection, statistics, or time windows. So, users need knowledge related to how they should define complex event patterns from open data sources. For instance, in Algorithm 2, the EPL sentences analyse the air quality parameters, specifically, parameters that should be analysed daily or annually, for instance, the $PM_{10}$ field should have an annual average value of less than or equal to 20 µg/m${}^{3}$ and a daily average of less than or equal to 50 µg/m${}^{3}$ (daily average).

#### Event Time Considerations

Usually, in order to carry out a successful analysis using Complex Event Processing, we should consider the time at which the event occurs. This is a natural issue when the events are caught in real-time, for instance, where a sensor provides data that are processed directly by Complex Event Processing or specific software. However, related to the open data sources, we should take into consideration that all data are first stored and later made available to be downloaded and processed. The use of these open data requires the execution of queries later on using data that have been produced earlier. As a consequence, we should identify how the event time should be analysed. Usually, this task requires the identification of the time-stamp field at the open data source. In other cases, a set of fields could be joined in order to suitably identify the time-stamp, for instance, supposing that an open data source includes *seconds*, *minutes*, *hours*, *days*, *months*, and *years* as fields to temporally identify each event. Time-stamp data allow analysers to suitably establish the temporal frame which enables the definition of complex patterns and analyses based on the time-stamp of when events occur.

In the previous example, the time-stamp was defined by the *SolarDate* attribute using the following template *“yyyy-MM-dd-hh:mm:ss”*. In Algorithm 2, the event patterns are defined based on time issues.

**Algorithm 2:** Example of EPL sentences working with window time.
1
**create schema** AirDataG (Station Float,Title String,Latitude Float,Longitude Float,SolarDate
2
String,SO2 Float,NO Float,CO Float,PM10 Float,O3 Float,dd Float,vv Float,TMP Float,HR Float,
3
PRB Float,RS Float,LL Float,BEN Float,TOL Float,MXIL Float,PM25 Float);
4
 
5
@Name("Air_PM10_Diary_Average")
6
**select**  a1.*
7
**from** AirDataG.win:time_batch(24 h).stat:uni(PM10) a1
8
**where** a1.average > 50;
9
 
10
@Name("Air_PM10_Anual_Average")
11
**select** a1.*
12
**from** AirDataG.win:time_batch(1 year).stat:uni(PM10) a1
13
**where** a1.average > 20;



### 3.3. Defining the Notification System When an Event Pattern Is Identified

Once an event pattern has been matched, the final users should be notified. Usually, this notification should be carried out by presenting the information on a log file or printing it out on the console. In addition, other notification mechanisms such as email, twitter or similar technologies could be explored. In our methodology, the notification phase defines several mechanisms, such as email, log files, Java Message Services (JMS), Twitter, and REST API services, that can be used to publish the complex analysis results. Thus, using JMS make it possible to generate a JMS message when an event patten has been matched, whereas using a REST to produce JSON or XML data improves the data reutilisation, offering the filtrated information obtained from the aforementioned analysis. Both allow users to generate value-added information which can be consumed by third tools or users.

From the point of view of open data sources, the offering of analysed results could help specific results to be found among a large amount of data. Besides, taking into account that our main goal is the analysis of near real-time open data sources, offering a notification based on the resources (for instance, JMS message or REST API) obtained helps users to suitably manage their email inbox and computer files, because the users do not store notification information which could be queried at a specific endpoint.

### 3.4. Executing the Continuous Queries on the Open Data Source

Using a CEP engine to analyse continuous open data allows users to join the best of two research areas: Complex Event Processing and open data. On the one hand, a CEP engine consumes a large amount of data in order to analyse them in a short period of time, thus consuming them in near real-time. On the other hand, open data offers well-structured information, for instance, based on public administration data.

Usually, CEPs are designed to consume stream data from several kinds of streams. For example, in *Esper CEP* [11], users can implement the data analysis using the *esperio-csv, esperio-db, esperio-amqp* extensions, among others, which make it possible to input CSV, DB, and AMQP data.

However, developers must write ad-hoc code in order to use these adapters/connectors, and, as a consequence, users require additional technical knowledge. From the point of view of open data sources where data are produced by using different data formats, friendly tools should be developed to improve their use with the goal of connecting, configuring, and deploying the CEP engine. From our point of view, the use of an Enterprise Service Bus (ESB) could help to achieve this goal.

In this sense, Several ESBs, such as MuleESB [57] and Apache Camel [58], make it possible to consume stream data based on different formats such as JSON, XML or similar. For this, the ESB engine usually has several adapters or connectors available. They could be connected with your favourite CEP. For example, Mule ESB allows access to both (i) different format files, such as CSV, JSON, XML, etc., and (ii) different communication protocols, such as HTTP, FTP, JMS, etc.

Thus, an Enterprise Service Bus (ESB) can be used to carry out continuous queries. Specifically, in our proposal, we used MuleESB [57], which facilitates the building of Service Oriented-Architectures (SOA) using common technologies (SOAP, REST, JMS or AMQP) and data transformation. In addition, from our point of view, the execution of continuous queries on open data sources could be carried out using cloud characteristics, such as easy deployment, infrastructure management, scalability, and so on. As a consequence, the application developed could be deployed in the cloud to improve scalability characteristics and pay per use. Besides, this deployment could be defined using current prominent tendencies, such as microservices deployed on containers, such as Docker or Kubernetes.

## 4. OpenData2CEP Tool: Design and Implementation Phases

**Figure 2:**
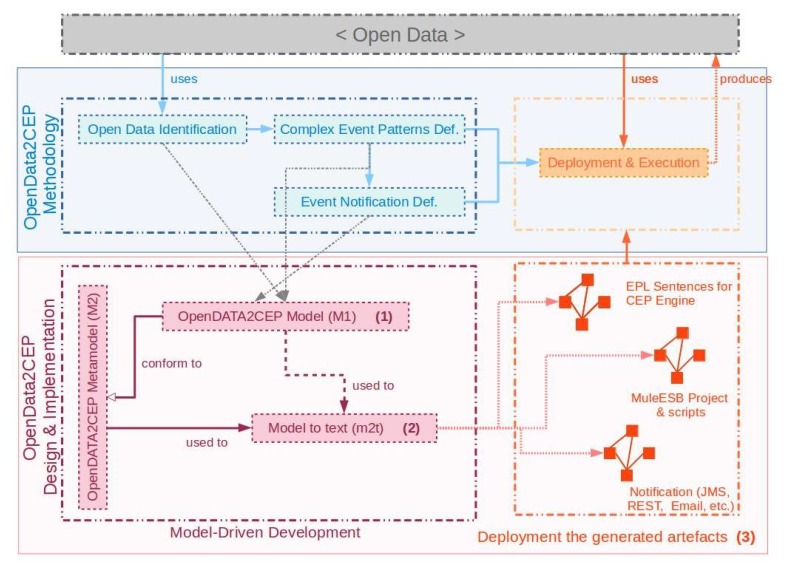
OpenData2CEP development vs. OpenData2CEP methodology.

In order to implement the aforementioned *OpenData2CEP* methodology a model-driven development approach was developed. Figure 2 shows the general overview of the OpenData2CEP model-driven approach where the main phases are identified: design (Figure 2 (1)), model-to-text transformation (Figure 2 (2)) and deployment and execution (Figure 2 (3)). The *design phase* (Figure 2 (1)) allows users to design the main configuration issues, such as the open data source identification or complex event pattern edition using models which help users to manage the open data and complex event concepts from a high abstraction level; the *model-to-text transformation phase* (Figure 2 (2)) implements a model-driven transformation, specifically, a model-to-text transformation, which makes it possible to generate the artefacts needed to deploy the system on a concrete ESB and CEP engine. Finally, the *execution phase* (Figure 2 (3)), allows users to deploy their specific queries on a concrete ESB and CEP engine.

**Figure 3:**
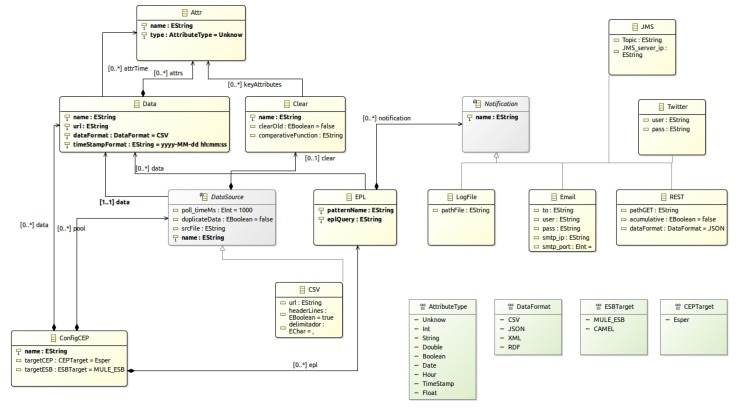
OpenData2CEP Metamodel.

Figure 3 presents the *OpenData2CEP* metamodel. Then, a model-to-text transformation is presented in order to obtain the ESB and CEP artefacts needed to carry out the complex event analysis. Finally, deployment issues are identified in order to facilitate the application deployment.

### 4.1. Design Phase: OpenData2CEP Metamodel

Figure 3 defines the *OpenData2CEP* metamodel, which represents the main concepts/entities and relationships needed to carry out complex analyses on near real-time open-data sources. Using model-driven development, users only need to know the concepts of this specific domain and their relationships, delegating the code generation and deployment in the specific model-to-text transformations developed ad-hoc.

The *OpenData2CEP* metamodel defines a *ConfigCEP* entity as the root element in the metamodel, identifying both *targetCEP* and *targetESB*, which define the final technology where the analysis will be executed. Currently, *targetCEP* only includes Esper [11] and *targetCEP* includes *MULE_ESB* [57]. However, the model-to-text transformation could be extended to allow other *targetCEP* and *targetESB*.

The *DataSource* entity defines the open data sources, including characteristics such as *poll_TimeMs* to identify how many milliseconds should pass before checking the data source again or whether *duplicateData* should be used to check if the data offered by the datasource should be analysed before to sending it to the CEP. The main goal of this concept is to specify whether duplicate data needs to be deleted. The *Data* entity makes it possible to define the specific characteristics of the data offered by the datasource: (i) *url* specifies the url where the data source could be queried, that is, where data could be downloaded; *DataFormat* specifies the data format, which could be one of the predefined *DataFormat* datatypes (CSV, JSON, XML or RDF). Users can define the datasource fields using the *Attr* entity. As has been mentioned, the data in the datasource should be frequently cleaned because two queries, for example, two consecutive GET HTTP methods on the *url* datasource, can produce similar results, including several already processed records. The *Clear* entity should be defined to configure the clean characteristics.

In order to define the complex analysis, users can define the *EPL* entity which wraps EPL sentences [59]. The *eplQuery* characteristic allows users to define complex patterns using the EPL pattern schema defined from the *Attr* entities (Users could learn how to write EPL sentences using the online analyser [60]).

Notification matters are defined using the *Notification* hierarchy which includes *LogFile*, *email*, *REST*, *JMS*, and *Twitter*. We focus on two of them, *REST* and *JMS*. The former makes it possible to publish the matched pattern using a specific REST infrastructure available in a *url*, while the latter makes it possible to publish each event pattern matched as an event by using JMS architecture. Both of them make it possible to describe an open-data source which could be used as input to other complex analyses.

Besides the *OpenData2CEP* metamodel, a graphical concrete syntax is developed to facilitate users’ modelling to conform to the *OpenData2CEP* metamodel. Specifically, final users can use the Eclipse Graphical Editor that has been developed from the *OpenData2CEP* metamodel by using the Graphical Modeling Framework (GMF) [61] and EuGenia [62]. In the following sections, several examples are modeled using this graphical editor.

### 4.2. Model-to Text-Transformation Phase. ESB and CEP Artefacts Code Generation

A model-to-text transformation allows users to obtain a complete Mule ESB project which can be deployed on the Mule ESB engine. To obtain these results, the model transformation generates both: (i) an ESB project code and artefacts (including the CEP engine configuration) and (ii) an ESB notification project code. This code has been generated from models conforming to the *OpenData2CEP* metamodel using Acceleo [63].

#### 4.2.1. ESB Project Code and Artefacts

**Figure 4:**
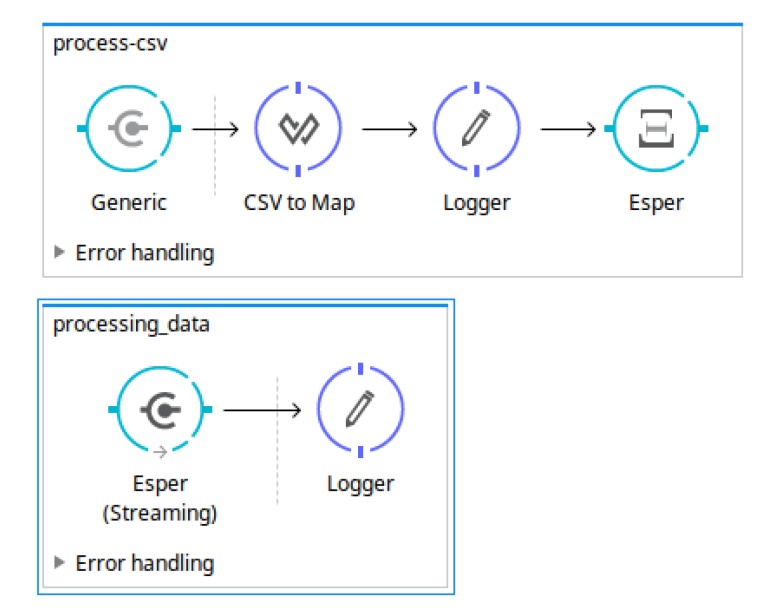
Example of Mule ESB workflow generated from an *OpenData2CEP* model.

The main workflow defined in the *MuleESB* project describes the steps used to carry out the data transformation needed to obtain suitable results. As can be observed in Figure 4, this workflow includes downloading the open data source, processing the data sources in order to delete the records already processed, and sending the records to the Esper Engine where they are analysed following the EPL sentences defined in the *OpenData2CEP* models. Finally, the events matched will send notification artefacts, for instance, emails, log files, JMS, etc.

Algorithm 3 shows an example of an *esper-config.xml* generated file which defines the event types that the Esper CEP engine should consume. Note that the generation of these files takes into account the *Data* elements defined in a specific *OpenData2CEP* model. As can be observed, the configuration file defines the event type for each *data* element defined as a *java.util.Map*. For instance, from a previously defined *data* element called *AirDataG*, the attributes *station, title*, *latitude* and *longitude*, among others, were generated as *map properties* at the *AirDataG java.util.Map*. This configuration file is used by Esper CEP to know the internal structure of each event type, that is, in our proposal, it corresponds with the structure of the open data source. Note that this structure is defined during the *design phase* when users define it in a specific *OpenData2CEP* model.

**Algorithm 3:** Example of Esper configuration file generated from the AirDataG OpenData2CEP model.
1
<esper-configuration
2
  xmlns="http://www.espertech.com/schema/esper"
3
  xsi:schemaLocation="http://www.espertech.com/schema/esper
4
  http://www.espertech.com/schema/esper/esper-configuration-2.0.xsd">
5
  <event-type name="AirDataG" >
6
    <java-util-map>
7
      <map-property name="Station" class="string"/>
8
      <map-property name="Title" class="string"/>
9
      <map-property name="latitude" class="float"/>
10
      <map-property name="longitude" class="float"/>
11
      <map-property name="SolarData" class="date"/>
12
      <map-property name="SO2" class="float"/>
13
      <map-property name="NO" class="float"/>
14
      <map-property name="CO" class="float"/>
15
      <map-property name="PM10" class="float"/>
16
      <map-property name="O3" class="float"/>
17
      ...
18
    </java-util-map>
19
  </event-type>
20
</esper-configuration>



#### 4.2.2. ESB Notification Project Code

Notification code is generated from the *OpenData2CEP* model. In this sense, most interesting strategies include the use of email notifications, REST API, and JMS notifications.

- **Email** notifications are implemented using Mule ESB Connectors which allows emails to be sent to previously modelled recipients using the SMTP protocol.
- **REST API** is generated in order to offer the matched event patterns.
  API REST is generated using the following schema. Note that each URL produces a JSON file with the data queried:

GET /analysis_project/epl_pattern/notification_name
GET /analysis_project/epl_pattern/notification_name?date=2016/11/10
GET /analysis_project/epl_pattern/notification_name?from_date=2016/11/10
- **Java Message Service (JMS)** notifications make it possible to publish a *topic* matched event pattern, and a publish/subscribe architecture is defined as a consequence. On the one hand, the Mule ESB could publish the matched pattern, while, on the other hand, any user interested in the results obtained from the event pattern analysis could subscribe to the *topic*. The message generated is a JMSTextMessage which includes a JSON record. By default, the *topic* is available in http://localhost:61616 where a *topic* named *topic.analysisProject.eplPattern.notificationName* is defined on *ActiveMQ* JMS Broker [64].

**Figure 5:**
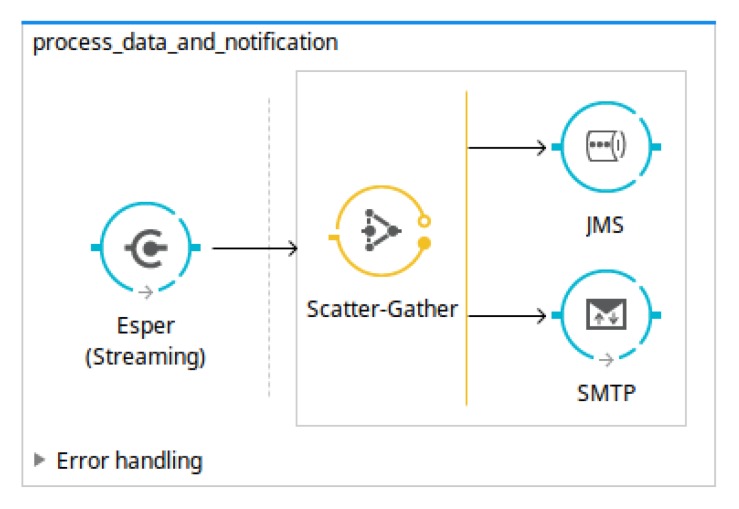
Example of Mule Enterprise Service Bus (ESB) flows generated from a Java Message Service (JMS) and email notification.

All notification strategies are generated from a model to Mule ESB flow, which generates all artefacts needed to deploy the whole analysis system using model transformation. Figure 5 shows an excerpt of the flows generated in order to publish the matched event pattern using JMS and email, that is, to generate the notification system.

## 5. Case Studies

Two case studies werecarried out using our methodology and tools in order to evaluate the aforementioned characteristics.

Thus, each case study is defined following the *OpenData2CEP* methodology, focusing on the *OpenDataCEP tools: design and implementation* phase based on model-driven development, as shown in Figure 2, which includes the *open data identification, complex event pattern definition and event notification definition* methodology phases Figure 2. Besides, from the point of view of the *implementation* phase, the model-to-text transformation is described for each case study.

The first case study is based on the Gijón open data website where air quality is measured. Our proposal includes continuously checking these data and analysing the air quality following the council norms. The second case study is an experiment developed using the earthquake data published by the U.S. Geological Survey open data website. For instance, earthquake data could be used to alert people in specific areas or to analyse earthquake magnitude tendencies.

### 5.1. Case Study: Air Quality

**Figure 6:**
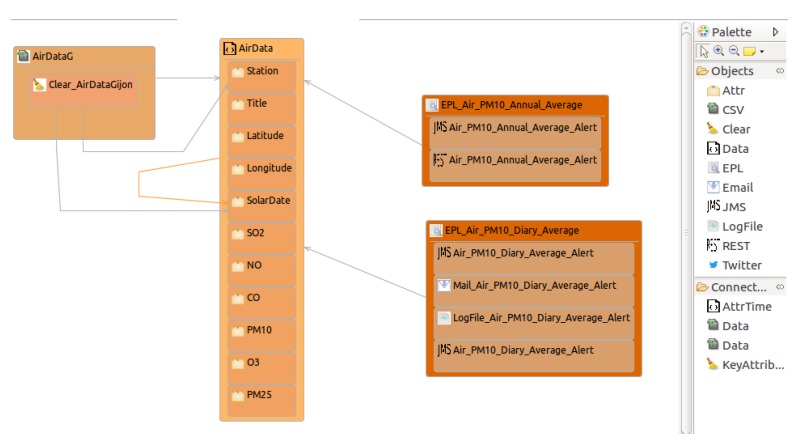
Case Study. AirDataG model conforming to the *OpenData2CEP* metamodel.

**Figure 7:**
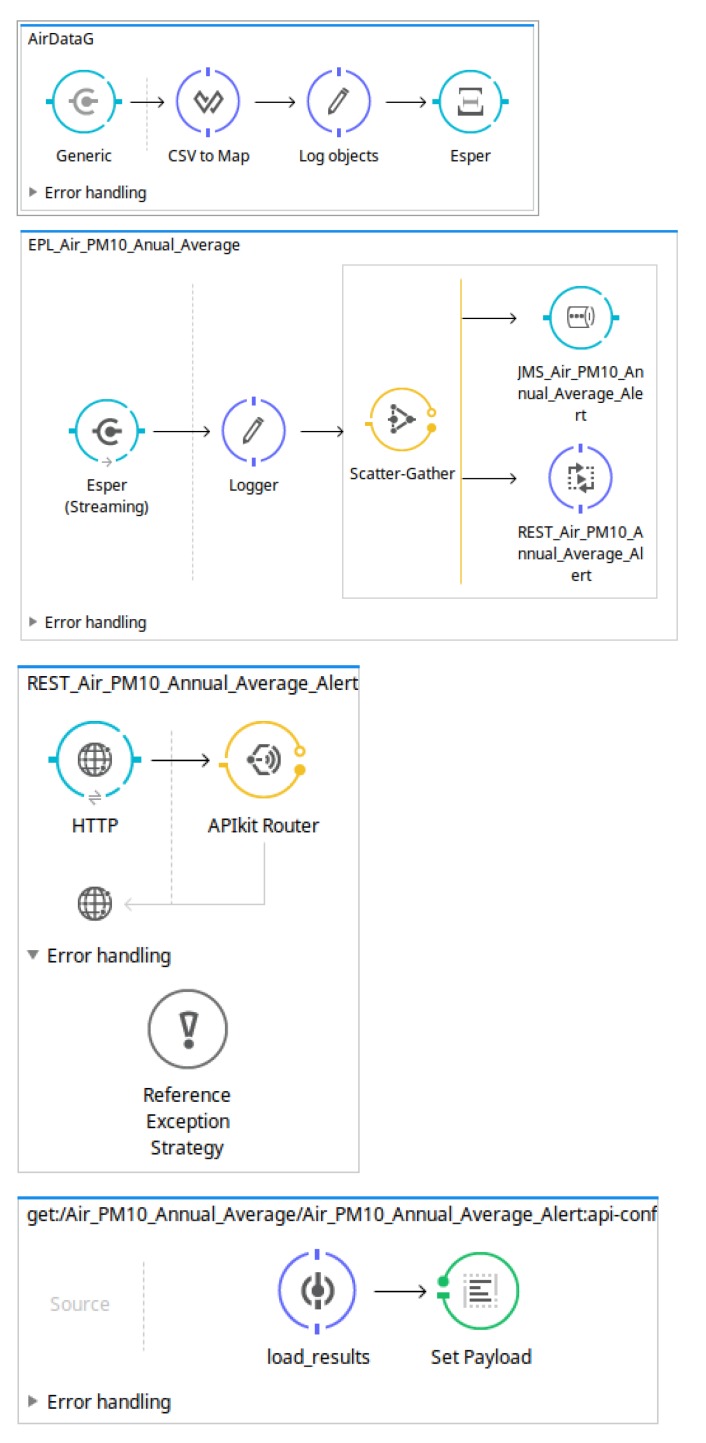
Example of main workflow generated at the Mule project from the AirDataG example.

This case study uses the open data produced by Gijón Council regarding air quality parameters (http://opendata.gijon.es/descargar.php?id=1tipo=CSV). For this case, we use our methodology and tools to define complex event analysis on this continuous data source:

- Design and implementation phase: design
  In Figure 6, we can observe the model *AirDataG* conforming to the *OpenData2CEP* metamodel. In this model, the needed entities have been defined to describe the main characteristics of the proposed analysis. Note that users could use the Eclipse Graphical Editor, which was developed as a concrete syntax for the *OpenData2CEP* metamodel. Specifically, a data source called *AirDataG* has been defined which manages *AirData* data elements defining several fields or attributes such as *station, title, latitude, longitude, solar date, etc.* Thus, the model defined includes the following main methodology phases: (i) data source identification; (ii) definition of complex event patterns and (iii) definition of matched event pattern on notification. Regarding the complex event pattern defined, as an example, both analyses are defined as follows: (i) the $PM_{10}$ field should have an annual average value of less than or equal to 20 µg/m${}^{3}$ and (ii) the $PM_{10}$ field should have a daily average value of less than or equal to 50 µg/m${}^{3}$. Note that these analyses include time windows which will be suitable managed by the CEP engine and can be defined using the EPL sentences presented below:

(i) EPL_Air_PM10_Diary_Average:
**select** a1.* **from** AirDataG.win:time(24 h).stat:uni(PM10)  a1 **where** a1.average > 50;
 
(ii) EPL_Air_PM10_Anual_Average:
**select** a1.* **from** AirDataG.win:time(1 year).stat:uni(PM10)  a1 **where** a1.average > 20;
  EPL sentences should be defined as properties on *EPL* entities, specifically at the *eplQuery* attribute. Then, as can be observed in Figure 6, notification issues are defined from each EPL sentence. For instance, for the EPL named *EPL_Air_PM10_Annual_Average*, both notification kinds have been defined: REST and JMS notifications.   
- Design and implementation phase: model-to-text transformation
  The artefacts obtained after model-to-text transformation allow users to deploy the whole analysis system on a MuleESB instance, including *Mule project*, *esper-config.xml*, *data input metadata for MuleESB*, *data output metadata for MuleESB* and *workflow.xml*, among others. The data flow is presented graphically in Figure 7. It includes a main *AirDataG* flow to address the events to the CEP engine (Esper). A second flow named *EPL_Air_PM10_Annual_Average* processes the matched event patterns, addressing them to specific notification flows. Specifically, in Figure 7, we can observe the REST API deployed in a flow named *REST _Air_PM10_Annual_Average_Alert*.
  Thus, notification issues are published following the model definitions (Figure 6). For instance, both REST and JMS notifications were defined from the EPL sentence named *EPL_Air_PM10_Annual_Average*.
  First, the REST API for the notification proposal (Algorithm 4) is generated using the RAML specification [65] and Apikit project [66] for integration with Mule ESB. Specifically, REST API defines the following urls for *AirPM10Alert _AnnualAverageNotification* notification, which are available through HTTP methods:

GET /AirDataG/AirPM10Alert_AnnualAverage/AirPM10Alert_Annual_Average_Alert
GET /AirDataG/AirPM10Alert_AnnualAverage/AirPM10Alert_Annual_Average_Alert?date=2016/11/10
GET /AirDataG/AirPM10Alert_AnnualAverage/AirPM10Alert_Annual_Average_Alert?from_hour=00:00:00
  Second, the JMS notification defined includes the properties *server* and *topic*. These properties can be used to subscribe a JMS client to the topic:

Server: http://localhost:61616
Topic: AirDataG.Air_PM10_AnnualAverage.AirPM10_Annual_Average_Alert

**Algorithm 4:** REST API generated defined by RAML to be deployed using the Apikit tool for Mule ESB.
1
#%RAML 0.8
2
---
3
title: AirDataG
4
version: 1.0
5
baseUri: http://localhost:8080/AirDataG
6
documentation:
7
  - title: Getting Started  AirDataG
8
    content:  |
9
    REST api notification for AirDataG.
10
 
11
/Air_PM10_Annual_Average/AirPM10_Annual_Average_Alert:
12
  displayName: AirPM10Alert_DiaryAverage
13
  get:
14
    description: Obtain information from a collection of AirDataG
15
    queryParameters:
16
      date:
17
        description: date
18
        type: string
19
        required: false
20
        example: 2016/10/10
21
 
22
      form_date:
23
        description: from_hour
24
        type: string
25
        required: false
26
        example: 00:00:00
27
    responses:
28
      200:
29
        body:
30
          application/json:
31
            schema: !include schemas/airdatag-schema-input.json
32
            example: !include examples/airdatag-example.json
33
      404:
34
        description: |
35
          Unable to find a AirDataG values



### 5.2. Case Study: Earthquake Data

The other case study that we developed to show our methodology and tools focuses on earthquake observations offered by the U.S. Geological Survey https://earthquake.usgs.gov/earthquakes/feed/v1.0/csv.php. These earthquake data are published in close to real-time, specifically, for the past hour, 24 h, 7 days, and 30 days in ATOM, KML, or text (csv) file formats. Specifically, the last hour earthquake data https://earthquake.usgs.gov/earthquakes/feed/v1.0/summary/all_hour.csv  allows users to carry out a continuous analysis. For instance, by using these earthquake data, users could analyse earthquake magnitude tendencies or issue alerts to people where earthquakes with a magnitude greater than a specific value will be reached.

**Figure 8:**
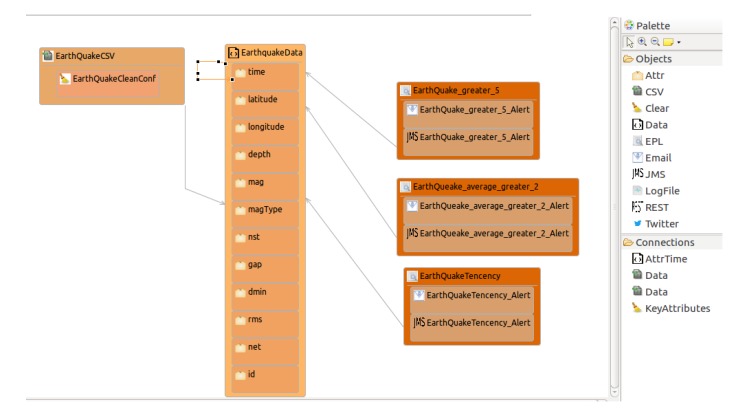
Case study: model of earthquake data analysis conforming to the *OpenData2CEP* metamodel.

**Figure 9:**
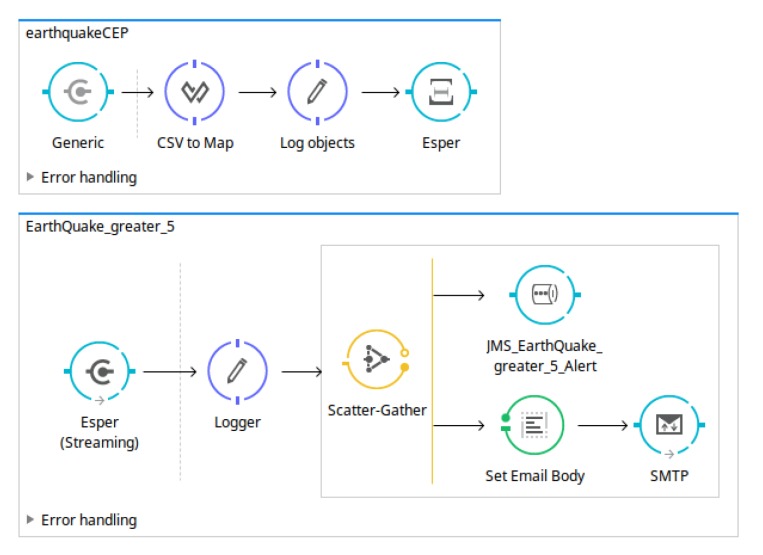
Example of Mule workflow generated in a Mule project from the earthquake data model defined in Figure 8.

- Design and implementation phase: design
  The open data source could be found at https://earthquake.usgs.gov/earthquakes/feed/v1.0/csv.php, where active earthquake data are updated continuously with a frequency close to real-time. Specifically, we use the last hour earthquake data here. Thus, the earthquake data include several items of information, such as *time, latitude, longitude, depth, mag, magType, nst, gap, dmin, rms, net, id, updated, place, type, locationSource, magSource, horizontalError, depthError, magError, magNst*, and *status*. For example, *latitude* and *longitude* mean the latitude and longitude values used to identify a place, while *mag* defines the earthquake magnitude on the Richter scale.
  Figure 8 shows the *OpenData2CEP* model for this case study, including the *Data, DataSource, ConfigCEP, Notifications*, and *EPL* entities. The model defined includes how to locate the data resource and the set of fields defined.
  Taking into account the fields identified at the open data source and defined in the model, users can define their own event patterns. For instance, a user can define several patterns based on earthquake data which could be useful for final users: (i) Alerts from earthquake magnitudes (mag) with values ≥5; (ii) Places (longitude and latitude) where the average earthquake magnitude (mag) was ≥2 in the last 5 days. (iii) Places (longitude and latitude) where the earthquake magnitude (mag) is incremental, starting from a magnitude value (mag) ≥2. Specifically, tree incremental earthquake magnitude values are identified.
  Note that a specific area could be defined by the user, for example, by indicating two latitudes/longitudes which build an area. In this sense, the event should be analysed by grouping the data by user areas.

(i)
@Name("EarthQuake_greater_5") **select**  * **from**  EarthquakeData **where** mag > 5;
 
(ii)
@Name("EarthQueake_average_greater_2") **select**  a1.* **from**
EarthquakeData.std:groupwin(longitude,latitude).win:time(5 day).stat:uni(mag) a1
**where** a1.average>2 and a1.datapoints≥2;
 
(iii)
@Name("EarthQuakeTencency")
**select** a1.longitude, a1.latitude, a1.mag, a2.longitude, a2.latitude,a2.mag, a3.longitude,a3.latitude,a3.mag
  **from pattern** [(**every** (a1 = EarthquakeData(a1.mag ≥1)
    -> a2 = EarthquakeData(a2.mag > a1.mag)
   -> a3 = EarthquakeData(a3.mag> a2.mag)
))]
**where** a1.longitude = a2.longitude  and a1.latitude = a2.latitude and a1.latitude=a3.latitude and a1.longitude=a3.longitude;
  From the point of view of notification issues, as can be observed in Figure 8, two types of notifications were defined: JMS and email notifications.
- Design and implementation phase: model-to-text transformation
  The artefacts obtained after model-to-text transformation allow users to deploy the whole analysis system on a MuleESB instance, including the *Mule project*, *esper-config.xml*, *data input metadata for MuleESB*, *data output metadata for MuleESB* and *workflow.xml* files, among others. The data flow is presented graphically in Figure 9.
  The notification code is generated from the model definitions (Figure 8), that is, JMS and email notifications are defined and are generated to be deployed with the following characteristics.
  The JMS notification defined includes the properties *Server* and *Topic* which are used to configure a JMS Connector for MuleESB:

Server: http://localhost:61616
Topic: earthquake.EarthQuake_greater_5.EarthQuake_greater_5_Alert
  For email notifications, the properties defined are *User, Pass, Server*, and *Port*:

User: user@unex.es
Pass: *****
Server: tajo.unex.es
Port: 995
  Thus, the code generated includes: (i) the MuleESB project; (ii) the Esper Mule workflow to analyse the input data; and (iii) the notification code which includes JMS and email Mule workflow.

## 6. Discussion

The use of model-driven development allows users to tackle technological complexity and manage heterogeneous technology from an abstract point of view. That is, applying software engineering approaches such as model-driven development allows focus to be on the important issues, such as the data analysis, rather than on technological troubles.

In this sense, model-driven development [17] focuses on raising the level of abstraction in software development by working with models instead of focusing on code. In this study, we proposed a metamodel named *OpenData2CEP* to guide the data analysis by generating the code artefacts needed to deploy the analysis data on a Complex Event Processing Engine.

Currently, available data is heterogeneous and varied, involving data on the environment, traffic, water, agriculture, economics, aero-space, public administration, society, education, culture and leisure, health, urban infrastructure, and security, among others. This information is published by open data proposals, as has been mentioned, such as https://usopendata.org or http://data.gov.uk. However, although open data is an emerging development area, it is not sufficiently visible for people because the data analysis is not within everybody’s reach. There are many open data sources that offer data through multiple open data formats, and their main motivation is to improve the data reutilisation. However, for society, the most important value is the data itself, that is, people want to improve their lives through data analysis if possible. In this sense, this approach tries to direct users towards data analysis by improving the methodologies and tools available to carry out these tasks.

Nowadays, complex-event analysis is needed to obtain added value from data and specifically for near real-time data such as those usually offered by open data catalogues. For this, our proposal includes the use of a complex event pattern engine which facilitates the execution of EPL sentences based on a concrete open data catalogue. In this sense, complex event correlations and event patterns can be defined to be applied on the open data sources which are continuously polled in order to maintain the input data near real-time.

### Limitations

In order to use our approach, users must know the EPL language so that they can define complex event patterns to obtain better results, thus increasing the learning curve of our approach. Nevertheless, other approaches, such as [51], make it possible to manage and define EPL languages using model-driven technologies, specifically, by using an EPL graphical editor auto-generated from event data definition.

Currently, our approach is limited to managing CSV formats because we have developed a prototype tool; however, we aim to include other widely used open data formats, such as XML and JSON. Then, the use of RDF data as an input will be evaluated because although RDF offers meaningful advantages related to linked data, it involves additional work which will be tacked in a further study.

## 7. Conclusions

Open data represents one of the main alternatives for democratising information. Generally, this information is generated from public administrations that develop their work using citizens’ taxes. Open data analysis should be improved through methodologies and tools that facilitate their use. Along this line of thought, this study presents a methodology and its model-driven approach. On the one hand, the methodology focuses on the main elements that should be taken into account in order to tackle the complexity of analysing near real-time open data sources. For this, adding a complex event processing engine decreases the time and effort needed to carry out the analysis. On the other hand, the model-driven implementation presented to carry out this methodology makes it possible to tackle the complexity of heterogeneous technology, such as data formats, complex event processing engines, Enterprise Service Buses, etc. Thus, the use of models to represent complex context like this makes it possible to focus on the main issues in the domain, delegating the generation of final artefacts needed to deploy the analysis system to model transformations.

For complex-event processing, the information is organised by events, which usually have a well-known structure based on specific data-types. For instance, in the previous example based on quality air data, each record has the same structure in several fields. However, when we work with linked data, each field in a record can be connected with external information, for example, by using an URI or relations with other concepts. Under these circumstances, defining a complex event pattern is more difficult; however, exploring the characteristics of linked data will help us to improve our proposal.

Another important further study will be focused on implementing a push notification, that could be integrated with a message platform such as Google Cloud Messaging [67], nimBees [68], etc.
